# Culture of the Aspirated Coronary Thromboembulus Specimen: A Peculiar Diagnostic Method for Infective Endocarditis

**DOI:** 10.1155/2013/607393

**Published:** 2013-02-27

**Authors:** Massimo Slavich, Andrea Fisicaro, Eustachio Agricola, Giovanni Coppi, Carlo Ballarotto, Alberto Margonato

**Affiliations:** ^1^Division of Cardiology, San Raffaele University Hospital, Via Olgettina 58, 20100 Milan, Italy; ^2^Division of Vascular Surgery, San Raffaele University Hospital, Via Olgettina 58, 20100 Milan, Italy

## Abstract

A 69-year-old man was admitted to our hospital for persistent fever, myalgias, articular pain, headache, and hypoaesthesia of the scalp. The clinical scenario was typical for giant-cell arteritis. During hospital stay, patient developed fugax amaurosis, stroke, and acute coronary syndrome. The definitive diagnosis of infective endocarditis, supported by transesophageal echocardiography, was confirmed only by culturing the material obtained during angiography and coronary thromboaspiration.

## 1. Introduction

Infective endocarditis (IE) is an infection of the cardiac endothelium, macroscopically seen as vegetations. The clinical manifestations are highly variable and often misleading, ranging from subtle symptoms to fulminant congestive heart failure with severe valvular regurgitation.

General and constitutional symptoms, like fever, fatigue, arthralgias, and myalgias, may be the only symptoms of IE for a long time. Laboratory findings often reflect a nonspecific inflammatory response, such as normochromic normocytic anemia, mild leukocytosis, elevated erythrocyte sedimentation rate (ESR) and C-reactive protein (CRP), and presence of rheumatoid factor and hypergammaglobulinemia. For these reasons, IE could be easily misdiagnosed as an immunologic disorder, such as rheumatoid arthritis, vasculitis, or collagen disease.

Systemic embolization occurs in 25 to 50% of cases; in fact, IE may present with amaurosis fugax, stroke, acute coronary syndrome, peritonitis or cold extremities. So, for these reasons, sometimes the diagnosis of IE can be very challenging.

## 2. Case Report

A 69-year-old man, with a history of gout and dyslipidemia treated with statins, was admitted to our hospital for the presence of persistent fever lasting for 20 days, with an evening peak of 38°C, associated with myalgia, diffuse articular pain, especially of the large joints, and frank arthritis of the right knee. He also referred recent new-onset headache and hypoaesthesia of the scalp.

Since a minimal diastolic murmur was occasionally discovered, the patient underwent to a transthoracic echocardiography one year before that documented a moderate aortic regurgitation.

On admission, the patient was in normal mental and haemodynamic state. The physical examination revealed only a mild aortic diastolic murmur. Temperature was 37.3°C, and routine blood tests showed CRP 101.6 mg/L, ESR 70 mm/hour, creatinine 1.42 mg/dL, fibrinogen 438 mg/dL, and hemoglobin 11.4 g/dL. Chest X-ray and ECG were normal. The patient indicated Levofloxacin treatment in the previous week.

During the hospital stay, blood tests for autoimmunity (ESR, ANA, ANCA, ASOT), wrist and hip X-rays, PPD skin test, and blood cultures were performed and all resulted negative. However, patient continued to complain of episodes of evening fever (peak temperature 37.5°C) despite a progressive reduction of the swelling in the right knee.

The rheumatologic consultation suggested a possible giant-cell arteritis; thus, the patient underwent temporal artery biopsy of the, which was negative, and steroid therapy was started (prednisone 0.6 mg/kg).

In the following days, the patient showed a slight benefit in the articular pain but developed an episode of amaurosis fugax. Considering the amaurosis secondary to refractory giant-cell arteritis, steroid dosage was increased, and antiplatelet therapy with aspirin was started.

One week following admission, the patient was found lying in bed, frankly stuporous, with a stable haemodynamic state and with no ischemic sign on the ECG. The neurological consultation documented a right hemiplegia syndrome. A cerebral CT scan was performed and no signs of intracranial mass or hemorrhage were shown, implying that the hemiplegia syndrome was of ischemic origin. In fact, the CT scan at 24 hours demonstrated a hypodense lesion of ischemic nature in the left parieto-occipital area at the grey-white junction, with no hemorrhagic component.

The day after, blood tests showed an increase in leukocyte count with neutrophilia that was considered secondary to steroid therapy; consequently the antibiotic therapy initially started was promptly stopped. Two days after, the patient indicated a typical dull thoracic pain, which radiated to the left arm. The ECG showed a lateral ST segment elevation. Due to the recent ischemic stroke, the patient underwent an urgent cerebral CT scan in order to rule out a possible hemorrhagic evolution of the stroke as this might have been a contraindication to an aggressive antiplatelet therapy. Once hemorrhage was excluded, the patient underwent primary percutaneous coronary intervention (PCI), with a loading dose of 600 mg of Clopidogrel and the administration of GP IIb/IIIa blockers during the procedure. The coronary angiography showed a complete occlusion of the very distal tract of the left anterior descending artery (LAD), while the remaining coronary arteries were completely free from any stenosis/atherosclerotic lesion. The interventionist only performed thromboaspiration of the thrombotic occlusion, without stent implantation.

In consideration of all the ischemic events, a possible relationship among all of them seemed reasonable. Since the embolization of vegetations from an IE could have explained acute myocardial infarction (AMI), amaurosis fugax and stroke, the filter containing the material obtained by means of thromboaspiration was sent to the bacteriology lab to be cultured in spite of preceding negative blood cultures. In the meantime, in order to confirm the suspicion of IE, a transesophageal echocardiography (TE) was performed, and three sets of peripheral blood cultures were taken again. Echocardiography showed, during diastole, the presence of a small dense formation of approximately 1 mm in diameter on the aortic valve, close to a moderate aortic regurgitation.

After two days, the thrombotic material showed growth of S. aureus, while just one out of three blood cultures resulted positive for S. epidermidis, clearly due to contamination. Steroid therapy was immediately stopped and a specific therapy with Vancomycin and Rifampicin was started, as suggested by the antibiogram.

## 3. Discussion

IE may be misdiagnosed with severe consequences. In our case, the clinical presentation and the age of the patient had led us to consider fever and arthromyalgia as rheumatologic in origin, being compatible with the diagnosis of Polymyalgia Rheumatica. Considering the strict link between Polymyalgia Rheumatica and giant-cell arteritis, headache, hypoaesthesia of the scalp, and amaurosis fugax were considered as rheumatologic also. Moreover, our patient seemed to reach the 3 out of 5 required criteria in order to make the possible diagnosis of giant-cell arteritis following the 1990 American College of Rheumatology classification criteria (Age > 50, ESR > 50 mm/hour, a new headache/dysesthesia of scalp) [1].

The distressing fact is that all these symptoms are also completely compatible with an embolizing IE. The angiographic finding of healthy coronary arteries and the evidence of a very distal occlusion of the LAD have strongly supported the idea that the AMI was independent from thrombosis on an unstable atherosclerotic plaque.

The peculiarity of our case report is that the final diagnosis of IE was made possible by cultures of embolic material collected during primary PCI. We underline the fact that because of previous antibiotic therapies, blood cultures always resulted negative. If we consider the Duke Criteria, we can notice that only with the culture of the thromboembolic material we were able to reach the necessary criteria (2 major criteria: the echocardiographic and the microbiological criteria) in order to make a definite diagnosis of IE [2].

In order to rule out IE, TE should probably have been performed earlier; however, the diastolic murmur, which was well known from his past medical history, was not highly suggestive, thus the rheumatological cause seemed more reasonable. To the best of our knowledge, this is the first case that IE diagnosis was achieved by the culture of the coronary PCI thromboembolic material.

Thrombus aspiration during primary PCI is indicated by myocardial revascularization guidelines [3, 4]. The main target of PCI is the restoration of the patency of the clogged epicardial vessel; however, distal microembolization can occur with consequent microvascular damage and thus lack of optimal myocardial reperfusion. Supported by the TAPAS trial results, which reported an improvement in indices of myocardial reperfusion and prognosis from routine use of manual thrombus aspiration before PCI or stent implantation, nowadays thrombus aspiration during primary PCI has been proposed to prevent distal embolization [5, 6].
